# Barrett’s Electro-Galvanometer

**Published:** 1848-07

**Authors:** 


*Barrett's Electro-Galvanometer.*—We have examined an Electro galvanic machine, for medical purposes, constructed under the direction of Dr. C. B. Barrett, of New York, which appears to us to possess some useful improvements. It resembles, in essential points, other machines similar in kind, but is more neatly made than any we have before met with. An armature for giving shocks, is attached to the machine. Dr. B. has added a metallic belt, by means of which shocks are transmitted directly through the abdomen, which are said to be useful in some cases of amenorrhœa, and constipation; also a pair of slippers, with metalic soles, in order that the circuit through the lower extremities may be more fully secured. We are informed by the agent that a few of the machines are left for sale at Mathews' medicine store. The price, without the appendages last mentioned, is $12 00. With the two appendages, $19 00.

